# Risk factors for therapy failure after surgery for perianal abscess in children

**DOI:** 10.3389/fsurg.2022.1065466

**Published:** 2022-12-15

**Authors:** Johannes Doerner, Rose Seiberth, Sakhavat Jafarov, Hubert Zirngibl, Lars Boenicke

**Affiliations:** Department of General and Visceral Surgery, Helios University Hospital Wuppertal, Witten/Herdecke University, Wuppertal, Germany

**Keywords:** anal fistula, surgery, children, perianal abscess, recurrence, pediatric proctology

## Abstract

**Purpose:**

The role of surgery in managing perianal abscesses in the pediatric population is debatable, and data on recurrence risk is rare. This study aimed to evaluate the efficiency of surgery for a perianal abscess in children and identify parameters that predict recurrence.

**Methods:**

We performed a retrospective review of all children younger than age 14 requiring surgery for a perianal abscess from 2000 to 2018.

**Results:**

Out of 103 enrolled patients, 27 (26%) had recurrent perianal disease. Recurrences appeared after a median of 5 months (range: 1–18 months), in 12 cases as perianal abscess and 15 cases as fistula *in ano*. Anal fistula probing was performed in 33% of all patients, of which 16 (15%) underwent fistulotomy. In univariate analysis, older age (*p* = 0.034), fistula probing (*p* = 0.006) and fistulotomy (*p* = 0.009) was associated with treatment success. History of perianal abscess, multilocal occurrence, and the presence of enteric flora in wound swabs was associated with treatment failure (*p* = 0.002, OR = 0.032). In multivariate analysis, anal fistula probing was independently associated with treatment success (*p* = 0.019, OR = 22.08), while the history of perianal abscess was associated with treatment failure (*p* = 0.002, OR = 0.032).

**Conclusion:**

Our study identified probing for fistula as a predictor of therapy success, while the history of perianal abscess was identified as a predictor of treatment failure. Therefore, in all children with perianal abscess, fistula probing and if present, fistulotomy should be performed.

## Introduction

The optimal management of perianal abscesses and fistula in children is controversial and differs from therapy recommendations in adults (1–3). In adults, about 90% of all cases with anal abscess and fistula *in ano* are assessed to have cryptoglandular etiology (4). An anal fistula and an anal abscess may occur alternately at the exact location and are therefore considered two clinical presentations of cryptoglandular infection (4, 5). In contrast, the pediatric disease affects almost exclusively boys, is only infrequently associated with sepsis, and may resolve spontaneously (6–8). While various approaches have been suggested to manage pediatric perianal abscess and fistula *in ano*, the optimal treatment is not well defined (9). While some authors favor conservative therapy with sitz baths with or without antibiotics (7, 10), others advocate incision and drainage followed by fistulotomy or fistulectomy (11–13). However, there may be a concern that fistula probing in pediatric settings could lead to iatrogenic fistula due to more delicate tissue in children. It is unknown which patients benefit from intensive fistula probing. The aim of this study was to evaluate the efficiency of surgery for perianal abscess in children and identify parameters that predict recurrence.

## Methods

### Study population

The local ethics committee approved the study protocol (AZ06/2018).

We identified all children aged 14 and below who underwent surgery for perianal abscess or fistula in our institution from January 2000 to December 2018. Data were derived from electronic patient records of our institution's medical database, including outpatient data. Demographic information, number and localization of lesions, clinical, laboratory, and microbiological data, usage and duration of pre-and postoperative antibiotics, abscess recurrences, fistula formation, and subsequent surgery for recurrences were analyzed. Children with anorectal malformations and inflammatory bowel disease were excluded from the study. The standard surgical procedure for perianal abscesses was incision and drainage under general anesthesia. Probing for fistula was generally only performed if the abscess was recurrent or a discharge of pus from the anal verge was identified during surgery. In the case of anal fistula detection, a fistulotomy was performed. In patients with the presence of phlegmon at abscess drainage, antibiotics were given postoperatively.

### Follow-up

The primary endpoint of the study was surgery for abscess recurrence or fistula. The latter was defined as nonhealing or persisting secretion of the wound three months after surgery. Follow-up information after discharge from the hospital was collected from outpatient records. In patients who had no contact with the hospital after discharge, the family physician or parents were contacted by telephone interview.

### Statistical analysis

Statistical analysis was performed with IBM SPSS Statistics Version 26 (IBM Inc., Armonk, United States). The primary outcome measure was abscess recurrence or fistula formation at follow-up. The success and failure groups' differences were evaluated using McNemar's test for categorical variables and the t-test for continuous variables. Logistic regression yielding odds ratios (ORs) was used to assess the significance of variables for success or failure. All significant risk factors found in univariate analysis were considered for inclusion in multivariate analysis (*p* < 0.05 for entry).

## Results

### Study cohort

**Figure 1:**
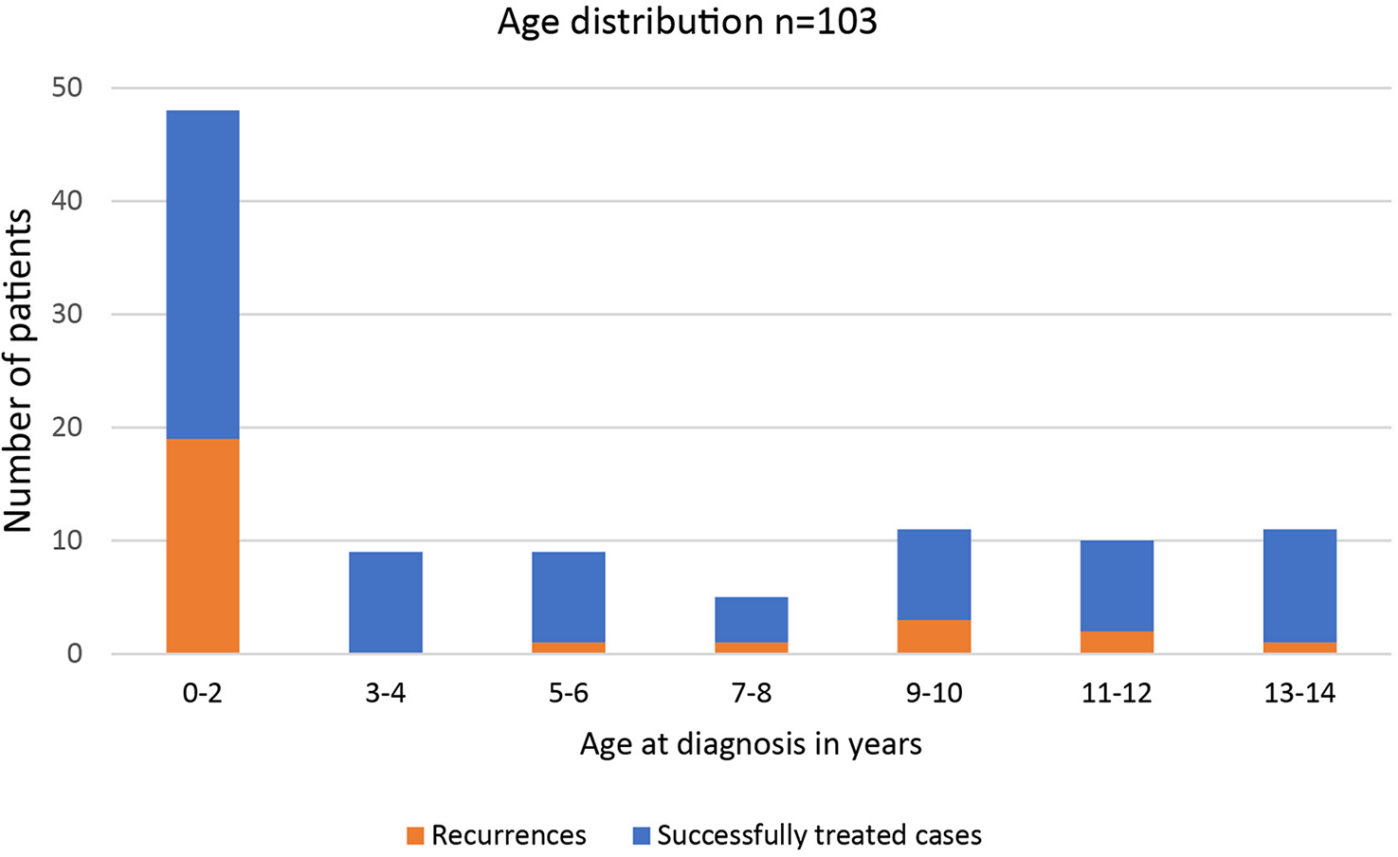
Distribution of incidence and outcome by age of onset of perianal abscess.

**Figure 2:**
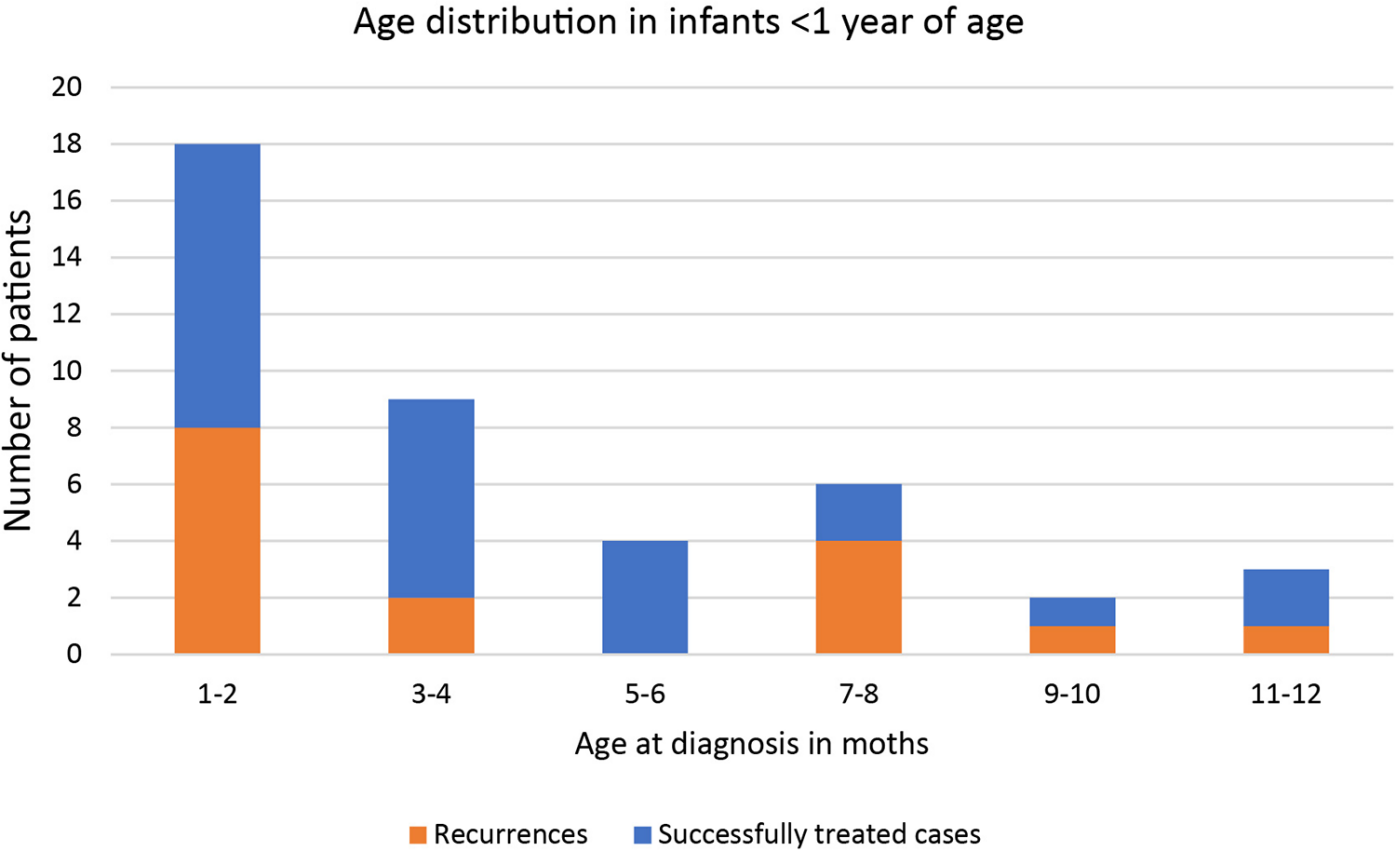
Distribution of incidence and outcome by month of onset of perianal abscess for infants under 1 year of age.

From January 2000 to December 2018, 129 children ≤14 years underwent surgery for a perianal abscess in our institution. Complete records were available for 103 consecutive patients. They were included in the study. The age distribution of the study cohort is shown in Figure 1. The median age was 2,28 years (range: 0 months to 14 years, interquartile range: 9.25 years). 46 (44.6%) were infants younger than two years, with most cases occurring during the first two months of life (Figure 2). The age distribution of the older children was homogenous. Patient history and preoperative demographical, clinical, laboratory, and microbiological characteristics are shown in Table 1. 89% of the study cohort were male. 26 (25%) patients had undergone conservative treatment, including antibiotics before surgery for a mean of 6.3(±3.4) days. 36 (35%) patients received antibiotics after surgery for a mean of 7.1(±3.3) days.

**Table 1:** Patient and disease characteristics related to success and failure of surgical treatment.

	All (103)	Success (76)	Failure (27)	Univariate *p*	Multivariate *p*^a^	OR
**Age**	**4.61** **±** **4.7**	**5.21** **±** **4.84**	**2.95** **±** **4.1**	**0**.**034**	n.s.	
Male Gender	89 (86%)	64 (84%)	25 (93%)	0.279	/	** **
Migration background	46 (45%)	38 (50%)	8 (29%)	0.069	/	
**Fistula Probing**	**33** (**32%)**	**30** (**39%)**	**3** (**11%)**	**0**.**006**	**0** **.** **019**	**22**.**08**
**Fistulotomy**	**16** (**15%)**	**16** (**21%)**	**0**	**0**.**009**	n.s.	
**History of perianal abscess**	**22** (**21%)**	**9** (**11%)**	**13** (**48%)**	**0**.**001**	**0** **.** **002**	**0**.**032**
Localization dorsal	33 (32%)	21 (28%)	12 (44%)	0.11	/	
Localization ventral	33 (32%)	24 (32%)	9 (33%)	0.868	/	
Localization lateral	37 (36%)	28 (37%)	9 (33%)	0.747	/	
**Multilocal**	**8** (**8%)**	**2** (**3%)**	**6** (**22%)**	**0**.**001**	n.s.	
**Enteric flora**	**87** (**84%)**	**61** (**80%)**	**26** (**96%)**	**0**.**049**	n.s.	
Elevated CRP, WBC	30 (29%)	24 (31%)	6 (22%)	0.363	/	
Body Temperature	37.21±0.6	37.25±0.6	37.11±0.7	0.377	/	
Size	15.90±9.6	16.61±10	13.8±7.3	0.207	/	
Antibiotics before surgery	26 (25%)	20 (26%)	6 (22%)	0.794	/	
Duration	6.3±3.4	6.17±3.3	8.6±3.1	**0**.**046**	n.s.	
Antibiotics after surgery	36 (35%)	25 (33%)	11 (41%)	0.467	/	

^a^ All significant variables in the univariate analysis were included in the multivariate analysis.

**Table 2:** Patient and disease characteristics according to type of abscess (primary vs. recurrent).

	All (*n* = 103)	Primary abscess *n* = 81	Recurrent abscess *n* = 22	Univariate *p*
Age	4.61±4.7	4.7±4,6	4.5±5.4	0.893
**Male Gender**	**89** (**86%)**	**67** (**83%)**	**22** (**100%)**	**0**.**036**
**Migration background**	**46** (**45%)**	**41** (**51%)**	**5** (**23%)**	**0**.**019**
Fistula Probing	33 (32%)	25 (31%)	8 (36%)	0.628
Fistulotomy	16 (15%)	12 (15%)	4 (18%)	0.702
Localization dorsal	33 (32%)	30 (73%)	9 (41%)	0.319
Localization ventral	33 (32%)	23 (28%)	10 (45%)	0.131
Localization lateral	37 (36%)	30 (37%)	7 (32%)	0.655
**Multilocal**	**8** (**8%)**	**3** (**4%)**	**5** (**23%)**	**0**.**003**
Enteric flora	87 (84%)	66 (81%)	21 (95%)	0.111
Elevated CRP, WBC	30 (29%)	21 (26%)	9 (41%)	0.173
Body Temperature	37.21±0.6	37.2±0.6	37.3±0.8	0.512
Size	15.90±9.6	16.9±10.0	12.4±7.0	0.054
Antibiotics before Surgery (days)	36 (35%)	26 (38%)	10 (23%)	0.248
Duration	6.3±3.4	7.1±2.6	6.7±3.5	0.661
Antibiotics after surgery (days)	36 (35%)	31 (32%)	5 (45%)	0.178
Duration	7.1±3.3	6.7±3.0	8.0±5.6	0.440
**Recurrence**	**27** (**26%)**	**14** (**17%)**	**13** (**59%)**	0.00005

### Abscess recurrence and fistula formation

**Figure 3:**
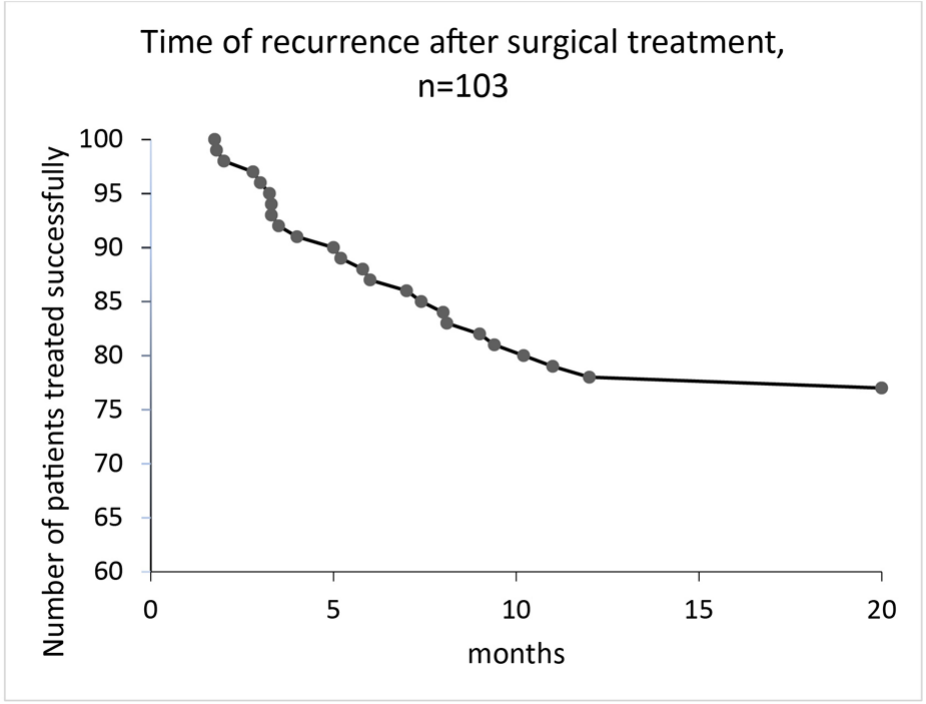
Time of recurrence after surgical treatment for perianal abscess in children, median follow-up was 22 (2–113) months.

**Figure 4:**
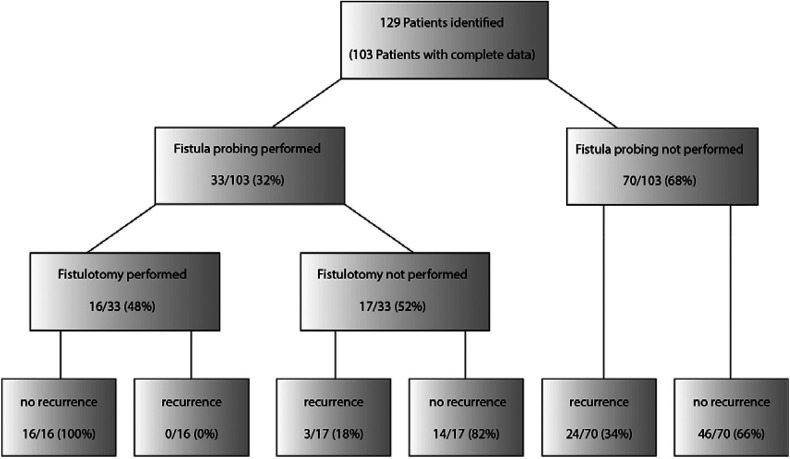
Summary of management and outcome of fistula probing for perianal abscess.

Follow-up after surgery was a median of 22 (2–113) months. Recurrent disease with the recommendation for surgery occurred in 27 patients (27%) after a median of 5 (1–18) months (Figure 3), in 12 cases as perianal abscess, and in 15 cases as fistula formation (Figure 4). One patient had recurrent disease more than 12 months after surgery.

### Rate of fistula detection

**Figure 5:**
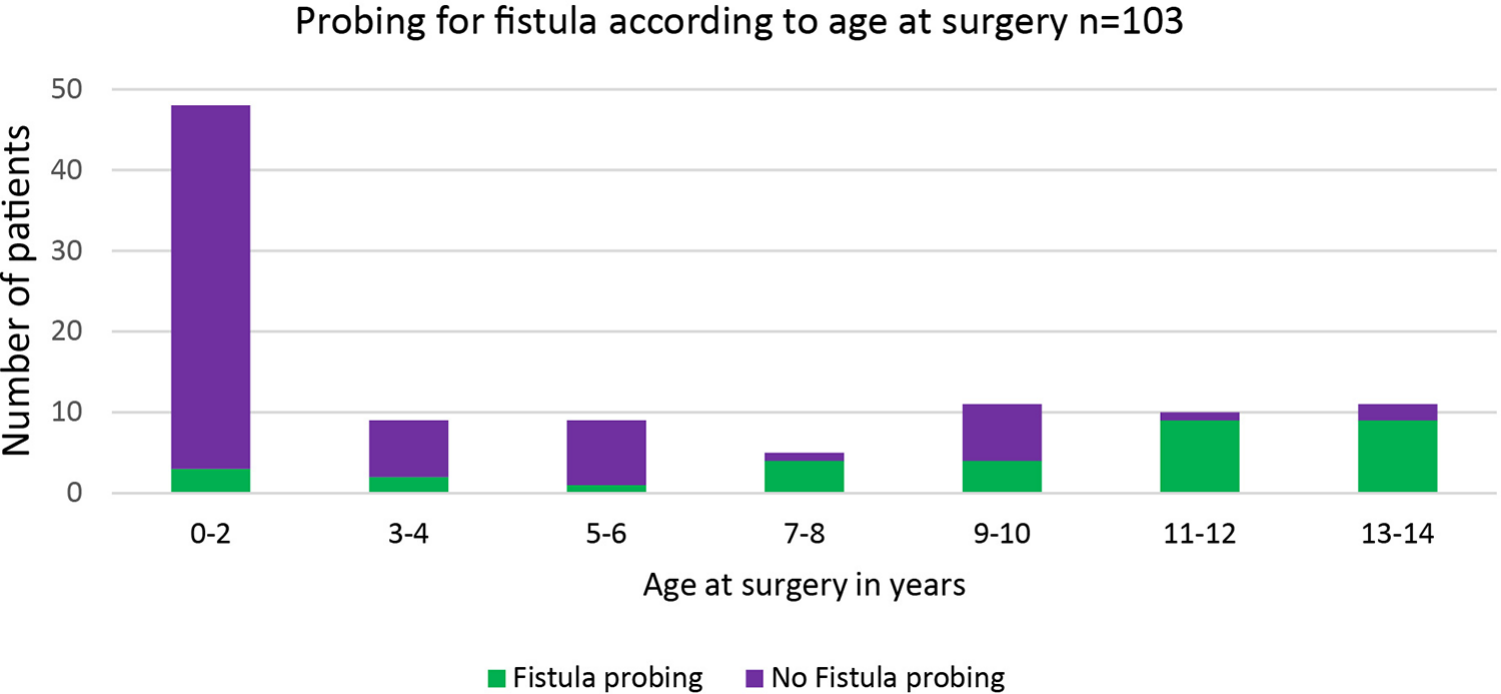
Execution of fistula probing relative to age at the time of surgery.

**Figure 6:**
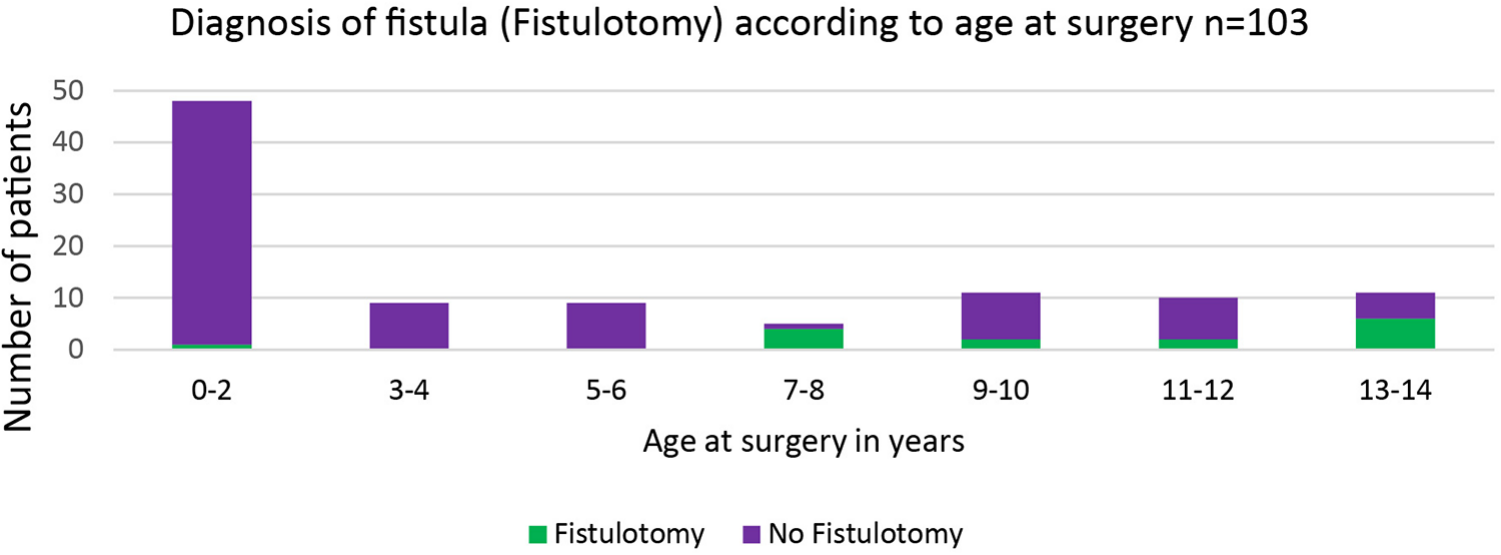
Diagnosis of fistula as defined by fistulotomy at time of surgery relative to age.

Fistula probing was performed in 33 (32%) patients, which resulted in fistula identification in 16 (16%) patients (Figure 4). In all patients with an identified fistula at surgery, a fistulotomy was performed, none of which had a recurrence. In all cases, fistulas had an inter- or low transsphincteric route. In our cohort, fistula probing, and identification were performed significantly more often in older patients (Figures 5, 6).

### Risk factors for recurrent disease

Univariate and multivariate analysis for risk factors for recurrent disease is shown in Table 1. The parameters age (*p* = 0.034), active fistula probing (*p* = 0.006), and fistulotomy (*p* = 0.009) were significantly higher in patients with therapy success. The parameters history of a perianal abscess (*p* = 0.001), multilocal localization (*p* = 0.001), and presence of enteric flora in wound swabs (*p* = 0.049) were significantly higher in patients with recurrent disease. The use of antibiotics, both pre-and postoperative, did not significantly influence the risk of recurrence. Following multivariate analysis, active fistula probing was an independent parameter for therapy success (OR = 22.08, *p* = 0.019) and history of perianal abscess for therapy failure (OR = 0.032, *p* = 0.002). Patient characteristics were analyzed according to the type of abscess (primary vs. recurrent, Table 2). In univariate analysis, male gender (*p* = 0.036) and multilocal abscesses (*p* = 0.003) were more frequently, and migration background (*p* = 0.019) was less frequently associated with recurrent disease. We found no difference in probing frequency for primary vs. recurrent disease (31% vs. 36%; *p* = 0.628). Therapy failure was associated with recurrent disease (primary: 17%, recurrent 59%, *p* = 0.00005).

## Discussion

Clinical characteristics and recommendations for managing pediatric perianal abscess and fistula *in ano* are heterogeneous in the literature.

This study aimed to identify risk factors for recurrence after surgery for a perianal abscess in children. In the present study, recurrence occurred in 27 out of 103 (26%) patients following surgical management of perianal abscess. Probing for possible fistula during the initial surgery was identified as a positive predictor for treatment success. In contrast, a history of a prior perianal abscess was a risk factor for recurrence.

The recurrence rate following surgery in our study is comparable to other studies in the pediatric population (14, 15) but lower than in the adult population, where recurrence as high as 40% has been described following incision and drainage (14, 15).

Recurrence may occur either as an abscess or a fistula *in ano* and is caused by persisting infection (4). Search for fistula and fistulotomy during abscess incision, and drainage prevent recurrence in the adult population (16). While there is concern that fistula probing in the pediatric setting may lead to iatrogenic fistula due to more delicate tissue in young individuals, when performed accurately, probing may identify a co-existing fistula as a potential source of recurrence. In our study, fistula probing was a significant predictor of therapy success. Interestingly, we found no difference in fistula probing between primary and recurrent abscesses. Concordantly with the multivariate analysis for therapy failure after surgical treatment which revealed fistula probing as a positive predictor for successful treatment, one can assume that the lack of consequent fistula probing in the recurrent abscess group may be a cause of undetected fistulas and secondary abscess formation. Recurrence was prevented in 16 cases (15%) by fistulotomy in the current study due to probing. This intriguing finding may suggest that the risk of creating an iatrogenic fistula during probing is minor. However, fistula probing in young individuals may be technically challenging, especially in infants. Knowledge of fistula routes is essential for correct probing, and probing must be cautiously performed. A relevant finding from our study was the history of perianal abscess as a risk factor for recurrence. This finding is similar to the adult population (17).

Many studies have reported a tendency for higher recurrence in infants compared to older children (9, 18, 19). In our study, younger age was associated with recurrent disease in univariate analysis but not multivariate analysis. This is in line with other studies, including the largest cohort reported so far by Gong et al. (6, 20). It remains unclear whether this is due to an alternative etiology or insufficient surgery, probably due to an overlooked fistula, especially in infants.

Christison-Lagay et al. report a significant reduction of fistula formation in patients receiving antibiotics (10). In our study cohort, antibiotics before or after surgery were not associated with the recurrence rate. This finding goes with many studies in adults, where antibiotics have not been shown to improve healing time or reduce recurrence rate (7–9, 19). In the present study, microbiological examination revealed the presence of enteric flora on wound swabs to be a risk factor for recurrent disease at univariate analysis but not at multivariate analysis. This is similar to findings in adults, where microbiological examination does not predict outcome and is thus not recommended (21, 22).

Nonetheless, our study demonstrates that probing for fistula could positively influence surgery outcomes for perianal abscesses in the pediatric population. A history of prior perianal abscess increases the recurrence probability following surgical management of perianal abscess in children. Therefore, all recurrent cases are best managed by specialists in colorectal surgery to reduce recurrence risk *via* cautious probing.

## Conclusion

Taken together, the risk of recurrent disease after simple abscess drainage in children, especially in individuals with a history of a perianal abscess, is noteworthy. Therefore, active probing for a fistula is strongly recommended, especially in cases with recurrence. Search for fistula and fistulotomy significantly decreases the risk of recurrence.

## Limitations of the study

This study has several limitations. The main limitation is the retrospective study design. Because the patients in this study were all seen in a hospital setting after surgical referral, it is possible that many perianal abscesses were treated medically without a surgical referral or surgically by the primary care provider using incision and drainage or needle aspiration. Therefore, we would advocate a prospective, randomized study to clarify the role of surgical treatment in this population.

## Data Availability

The original contributions presented in the study are included in the article/Supplementary Material, further inquiries can be directed to the corresponding author.
